# Toward a deeper understanding of dengue: novel method for quantification and isolation of envelope protein epitope-specific antibodies

**DOI:** 10.1128/msphere.00961-24

**Published:** 2025-04-11

**Authors:** Sokchea Lay, Candice Bohaud, Sopheak Sorn, Sreymom Ken, Felix A. Rey, Kevin K. Ariën, Sowath Ly, Veasna Duong, Giovanna Barba-Spaeth, Heidi Auerswald, Tineke Cantaert

**Affiliations:** 1 Immunology Unit, Institut Pasteur du Cambodge, Pasteur Network 533891, Phnom Penh, Cambodia; 2 Epidemiology and Public Health Unit, Institut Pasteur du Cambodge, Pasteur Network 533891, Phnom Penh, Cambodia; 3 Virology Unit, Institut Pasteur du Cambodge, Pasteur Network 533891, Phnom Penh, Cambodia; 4 Unité de Virologie Structurale, Institut Pasteur, Université Paris Cité, CNRS UMR3569 129771 , Paris, France; 5 Virology Unit, Department of Biomedical Sciences, Institute of Tropical Medicine 37463 , Antwerp, Belgium; 6 Department of Biomedical Sciences, University of Antwerp 573668, Antwerp, Belgium; University of Maryland School of Medicine, Baltimore, Maryland, USA

**Keywords:** dengue virus, antibody response to dengue, antibody-dependent enhancement, flavivirus anti-envelope antibodies

## Abstract

The dengue viruses (DENV) envelope (E) protein is the main target of the antibody (Ab) response. Abs target different epitopes on the E-protein, including sE-dimer, E domain III (EDIII), and fusion loop (FL). Anti-EDIII Abs are mainly serotype-specific, whereas anti-FL Abs can induce antibody-dependent enhancement (ADE) *in vitro*. Abs targeting sE-dimer epitopes can cross-neutralize different DENV serotypes. However, the involvement of each Ab subset in disease pathogenicity and/or protection remains unclear. We aimed to optimize the quantification and purification of DENV E-protein epitope-specific Abs from human samples. C-terminal biotinylated DENV2 E recombinant proteins (EDIII, soluble E [sE], and sE-dimer) were coupled to color-coded magnetic microspheres for a multiplex immunoassay (MIA), testing different antigen concentrations. Assay performance was evaluated using well-characterized anti-DENV monoclonal antibodies (mAbs) and total IgG from DENV seronegative and seropositive human plasma. Specific FL epitopes were blocked with mouse mAb clone 4G2 to quantify anti-FL- and sE-dimer-specific Abs, measuring antigen-antibody reactions as median fluorescence intensity (MFI). For isolation of E-protein epitope-specific antibodies, sE-proteins were conjugated to streptavidin resin beads. Total IgG from human plasma was incubated with immobilized EDIII to elute anti-EDIII Abs. The flow-through was incubated with sE-dimer resin beads to elute sE-dimer specific Ab enriched fraction, and the flow-through was applied to immobilized sE to elute anti-FL Abs. In conclusion, we have developed a serological assay to detect E-protein epitope-specific Abs in DENV-infected humans. Additionally, we successfully isolated anti-EDIII, anti-FL, and an enriched fraction of sE-dimer specific Abs from human samples.

**IMPORTANCE:**

The development of effective dengue virus (DENV) vaccines has been hampered by limited insights into the immunological mechanisms of protection. Our study addresses this gap by introducing a refined multiplex microsphere-based immunoassay (MIA) to quantify and isolate antibodies (Abs) targeting specific E-protein epitopes, such as E domain III (EDIII), the fusion loop (FL), and the sE-dimer specific Abs. This method provides detailed epitope-specific Ab profiling with high sensitivity and requires minimal sample volumes. The ability to isolate specific Ab subsets from human plasma also enables detailed investigations into their roles in protection or pathogenesis, paving the way for more effective dengue interventions.

## INTRODUCTION

Dengue is the most important vector-borne viral disease in humans, with half of the world’s population living in endemic areas (1). Currently, there is no specific treatment available, except for supportive care and careful monitoring of blood parameters and fluid loss. Dengue virus (DENV) belongs to the *Orthoflavivirus* genus of the *Flaviviridae* family. Viruses in this genus are antigenically highly conserved, leading to broad cross-reactivity. DENV comprises four distinct serotypes, DENV1 to DENV4, that co-circulate in the same endemic areas and are transmitted by *Aedes* mosquitoes (1, 2). DENV is a single-stranded, positive-sense RNA virus which consists of seven non-structural proteins and three structural proteins: capsid (C), envelope (E), and pre-membrane (prM). The maturation by furin processing of prM is required to yield infectious particles, which contain M. The ectodomain of the E-protein is composed of three structured domains: envelope domain I (EDI), domain II (EDII), and domain III (EDIII). The fusion loop (FL) peptide is located in EDII and is a key functional element of E, important to drive membrane fusion of the virus particle with the host cell endosomal membrane (3, 4). E-proteins form homodimers that cover the entire surface of DENV particles in a herringbone pattern. Epitope exposure at the surface of DENV particles is dependent on the maturation state (prM content) and the temperature (“breathing” process), exposing different epitopes in different virus states (5, 6).

A primary DENV infection induces potent protective, long-lasting antibodies (Abs) against homotypic re-infection (with the same serotype). In contrast, cross-reactive Abs binding and neutralizing heterologous serotypes are short-lived with a half-life ranging from several months to 3 years (7). DENV-specific Abs can neutralize the virus after homotypic re-infection but can also lead to more severe disease in a process of antibody-dependent enhancement (ADE) after heterotypic infection (1, 8–10). The functionality of the Abs depends on their titer, affinity, avidity, their targeted epitopes, and isotype (11, 12). The Ab response to DENV is mainly directed to the E-protein, including the well-characterized FL epitope, which is highly conserved among all flaviviruses (13). The FL peptide is immunodominant and usually buried at the interface of the E-dimers in mature particles; however, it is exposed in breathing particles (4). Monoclonal anti-FL Abs generally cross-react among the DENV serotypes, are weakly neutralizing, and can promote ADE *in vitro* (14, 15) and *in vivo* in animal models (16), but extensive studies in humans are lacking. In contrast, Abs targeting the structural E-dimer epitope (EDE) are very potent and broadly cross-neutralizing, with some anti-EDE monoclonal Abs (mAbs) even neutralizing Zika virus (ZIKV) (6, 17), another important closely related *orthoflavivirus*. EDI- and EDII-specific Abs are less effective and have greater cross-reactivity against other serotypes, while EDIII-specific Abs are serotype-specific and have a low potential to induce ADE (18–20).

Abs interfere with DENV infection and/or kill infected cells throughout several mechanisms: they prevent virus attachment to the cell surface, they interrupt viral membrane fusion (21), and/or trigger Fc-mediated effector functions by interacting with several epitopes of the E-protein (22). To understand the epitope specificity involved in neutralization and/or ADE, several studies have characterized the functionality of epitope-specific Abs by using mAbs isolated from dengue patients (6, 17, 23–25).

The neutralization and ADE potential of plasma depleted of Abs binding to either EDIII or soluble E (sE) has also been measured, where sE-depletion showed a decrease in neutralization capacity; depletion of EDIII-reactive Abs had little effect on neutralization or ADE in some studies; while in other studies, depletion of EDIII-reactive Abs resulted in lower neutralizing capacity (26–28). However, the natural conformation of the DENV E-proteins in a pre-fusion dimer (3, 29) presents a challenge in characterizing the functionality of Abs targeting FL and sE-dimer epitopes in their capacity for neutralization, ADE, and/or Fc-mediated effector functions (30).

Several serological tests have been developed using recombinant E-protein, inactivated virions, or virus-like particles. Enzyme-linked immunosorbent assays (ELISAs) are the primary serological tests for detecting anti-DENV Abs; however, they often lack sufficient sensitivity and specificity, and they are unable to distinguish Abs targeting different E-protein epitopes (31). Virus neutralization tests are considered the gold standard serological test to detect neutralizing Abs due to their high specificity in determining DENV serotype-specific Abs. However, these *in vitro* assays are technically challenging, difficult to interpret after multiple heterotypic infections, time-consuming, not amenable to high-throughput testing (32), and are unable to detect E-protein epitope-specific Abs.

These issues with traditional serological assays spurred the development of cost-effective, flexible, sensitive, and high-throughput methods for simultaneous detection of different Abs using multiple antigens (33, 34). The development of multiplex microsphere-based immunoassays (MIAs) allows the simultaneous quantitative detection of multiple analytes in a single biological sample, e.g., measuring different subsets of Abs targeting different epitopes. Therefore, we hypothesized that a MIA could be utilized to characterize DENV E-protein epitope-specific Abs in human plasma samples in order to characterize more in depth the humoral immune response to DENV E-protein.

In this study, we delineate a purification strategy for the different DENV E-protein epitope-specific Ab subsets (anti-FL Abs, anti-EDIII Abs, and sE-dimer specific Abs) and the quantification of these subsets using a multiplex MIA. These well-characterized subsets can be utilized for subsequent downstream applications for a deeper understanding of the functionality of the humoral immune response after DENV infection.

## MATERIALS AND METHODS

### Human plasma and monoclonal antibodies

DENV seropositive and seronegative human plasma samples from the DENTHOM project, and well-characterized anti-E human mAbs were used to validate our assays. As positive human controls, plasma samples collected from three individuals at day 10 after confirmed DENV2 secondary infection were used. Absence of serological evidence of DENV infection was confirmed by dengue IgG Indirect ELISA (Panbio, following manufacturer’s instruction) in three negative control samples. Rituximab, an anti-human CD20 mAb, was in-house produced as previously described (35). Furthermore, 1A5, a humanized mAb specific for the FL in EDII was produced by GenScript Biotech based on the published sequence (36). The mouse mAb clone 4G2 was purchased from GenTex International. The EDE-specific mAb clones C8 and C10, and the EDIII-specific mAb clone 1A1D-2 were in-house produced as described before based on publicly available sequences (17, 37).

In addition, we included DENV cases identified among hospitalized children presenting with acute dengue-like illness in a cohort at Kampong Thom and Siem Reap provinces (DENTHOM project). The patients were recruited at Kampong Thom Provincial Hospital, Baray-Santuk and Stuong district hospitals (Kampong Thom province), and at Jayavaraman VII Hospital (Siem Reap province) between 2022 and 2024. Plasma samples were collected 4–6 days after onset of symptoms . Dengue infection and immune status were confirmed as previously reported (38, 39). Only individuals undergoing secondary DENV2 infection were included.

### Total IgG isolation from human plasma

Human total IgG was isolated from plasma of both DENV seropositive and seronegative individuals using affinity chromatography (Protein G Sepharose 4 Fast Flow, GE Healthcare). The plasma samples were diluted at a 1:1 ratio with 1× phosphate-buffered saline (PBS) containing protease inhibitor (Roche). Protein G Sepharose beads were washed with 1× PBS (Thermo Fisher Scientific) and incubated with the pre-diluted plasma samples on a rotator for 1 hour at room temperature (RT). Following incubation, the beads were washed with 1× PBS, and IgG fragments were eluted using Pierce IgG Elution Buffer pH 2.8 (Thermo Fisher Scientific). Elution buffer was neutralized with Tris 1 M, pH 8 (Sigma-Aldrich) immediately after elution. The concentration of total IgG was determined using the Bradford assay (Bio-Rad).

### Recombinant proteins

DENV E recombinant proteins, namely EDIII (19 kDa), sE (50 kDa), and stabilized sE-dimer (100 kDa) proteins were produced, based on sequences of Cambodian DENV2 (GenBank accession number AHG23166) isolate, in *Drosophila* S2 cells as previously described (30, 40), with the addition of a His-tag, an Avi-tag, and a double Strep-tag at the C-terminus. Two mutations were introduced in the E-protein sequence obtained from the Cambodian DENV2 isolate (Leu107Cys and Ala313Cys) to generate two disulfide bonds which contain the sE in a dimeric form stabilizing the EDE epitope using the same strategy described before (30). The proteins were site-specifically biotinylated with the BirA biotinylation kit (GeneCopoeia) following the manufacturer’s instructions. Excess biotin molecules were removed using a cellulose membrane filter (Amicon Ultra 3 kDa and 30 kDa, Merck Millipore).

### Antigen coupling to MIA beads

Different color-coded magnetic microspheres (beads) (Bio-Plex Pro Magnetic COOH Beads, Bio-Rad) were coupled at RT to the DENV2 recombinant proteins (EDIII, sE, and sE-dimer), or streptavidin protein. The coupling was performed using the commercial Bio-Plex Amine Coupling Kit (Bio-Rad) following the manufacturer’s instruction manual. Briefly, 1.25 × 10^6^ beads were vortexed thoroughly for 1 minute and washed twice with the bead washing buffer using a magnetic separator. The beads were resuspended in 80 μL of bead activation buffer, and 10 μL of 50 mg/mL N-hydroxy-sulfosuccinimide sodium salt (Sulfo-NHS; Sigma-Aldrich) and 50 mg/mL 1-ethyl-3-(3-dimethyl-aminopropyl) carbodiimide hydrochloride (EDAC; Sigma-Aldrich) were added. The suspension was vortexed and then incubated in the dark on a rotator for 20 minutes at RT. Afterwards, the activated beads were washed with 200 μL of 1× PBS pH 7.4 (Bio-Rad). The activated beads were resuspended in 300 μL 1× PBS and 2–10 μg of recombinant protein or streptavidin was added to the activated beads. The suspension was incubated in the dark on a rotator for 2 hours at RT. The coupled beads were washed with 500 μL of 1× PBS, re-suspended in 250 μL blocking buffer (Bio-Rad), vortexed, and then incubated for 30 minutes in the dark on a rotator. The supernatant was removed, and the coupled beads were resuspended, washed with 500 μL storage buffer (Bio-Rad), vortexed, and finally resuspended in 150 μL storage buffer. The concentration of coupled beads was determined by using the Countess II FL Automated Cell Counter and stored at 4°C in the dark.

### DENV E-protein epitope-specific IgG detection via MIA

Before usage, the beads coupled to the DENV2 antigens (EDIII, sE, sE-dimer) were vortexed for 30 seconds and diluted to 2,500 beads/100 μL in assay buffer (1× PBS, 2% fetal bovine serum [FBS, Gibco], 1% bovine serum albumin [BSA, Sigma-Aldrich], and 0.1% Tween-20 [Bio-Rad]). Subsequently, 1,250 beads of each recombinant protein were added to each well of a 96-well flat-bottom plate (BioPlex Pro, Bio-Rad). In order to block the FL epitope, 0.5 µg of mouse mAbs clone 4G2 (GeneTex International Corp) was added to sE-protein containing wells. After five washes, the serially diluted total IgG from human samples or anti-DENV E mAbs were added to the beads. Next, the beads were incubated for 1 hour at RT in the dark on a plate shaker at 450 rpm. The bead-antibody complexes were washed five times with 150 μL washing buffer (1× PBS and 0.1% Tween-20). Subsequently, 0.1 µg R-phycoerythrin AffiniPure goat anti-human IgG (Fisher scientific) was added, and the complexes were incubated again for 1 hour at RT in the dark on a plate shaker at 450 rpm. To visualize the binding of mouse mAb clone 4G2 to the beads, PE goat anti-mouse IgG were used. Following five additional washing steps with washing buffer, the beads were re-suspended in 1× PBS, 1% BSA. The complexes were analyzed with the BioPlex-200 system (Bio-Rad) to quantify the amount of bound antibodies to each antigen. At least 50 events were analyzed for each antigen, and the results were expressed as median fluorescence intensity (MFI) per 50 beads. Relative abundance of 4G2-like Abs (representative of anti-FL loop Abs) is measured by subtracting the MFI signal of Abs bound to sE pre-incubated with mouse mAb clone 4G2 from the total signal of Abs bound to sE. Similarly, sE-dimer specific Abs were quantified by subtracting the MFI signal of Abs bound to sE pre-incubated with mouse mAb clone 4G2 from the Abs bound to sE-dimer protein. DENV-E mAbs were measured in two independent experiments, and data represent the mean ± standard error of the mean (s.e.m.).

### Purification of DENV E-protein epitope-specific IgG from human plasma

**Fig 1:**
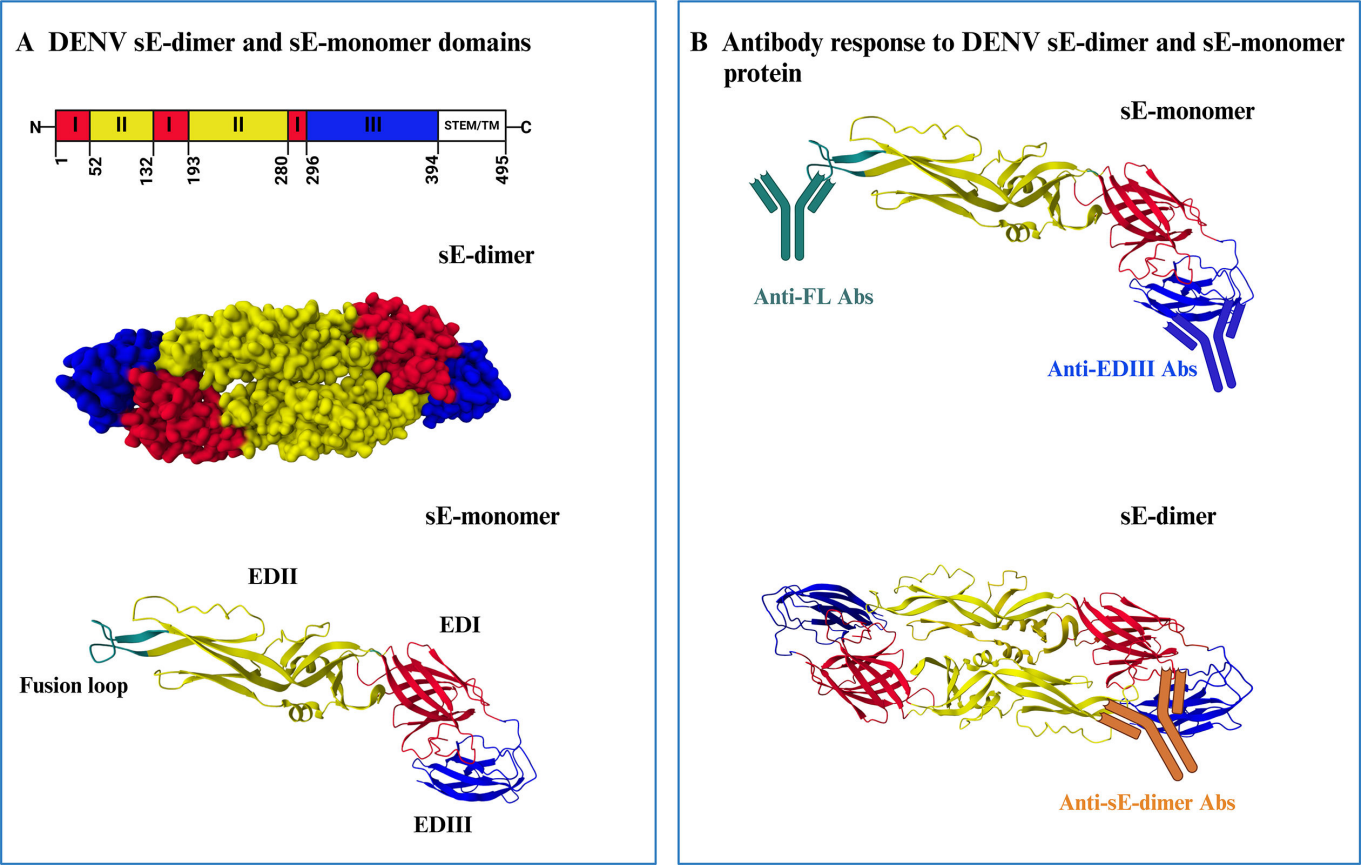
DENV E-protein domains and antibody response. (A) Structural organization of DENV E protein (PDB:7A37) in soluble E (sE)-dimeric and sE-monomeric form. Each monomer consists of three ecto-domains: EDI (red), EDII (yellow), EDIII (blue), and fusion loop (FL) (green) (3, 4). (B) Antibodies targeting different epitopes on DENV E-protein: anti-EDIII Abs (blue) bind to both sE-monomer and sE-dimer due to the availability of the EDIII epitope on both forms. Anti-FL Abs (green) bind only to sE-monomer as the FL is not exposed in the sE-dimer. sE-dimer specific Abs (orange) bind to quaternary epitopes and hence only bind to sE-dimer protein.

C-terminal biotinylated recombinant E-derived proteins were conjugated with streptavidin resin beads (Pierce, Thermo Scientific) by incubation for 1 hour at RT on a rotator. The coated beads were then used for the purification of DENV E-protein epitope-specific Abs (Fig. 1). First, total IgG derived from human plasma or mAbs was incubated with EDIII-coated beads on a roller for 1 hour at RT. Subsequently, the IgG-bead suspension was transferred to a chromatography column and centrifuged at 1,500 rpm for 10 seconds to remove unbound Abs (fraction 2). The beads were washed with 500 µL of 1× PBS for 10 times, and Pierce IgG Elution Buffer (Thermo Scientific, adjusted with hydrochloric acid to obtain pH 2.3) was added into the column and centrifuged at 1,500 rpm for 10 seconds to collect anti-EDIII-specific Abs (fraction 1). Here, the pH was immediately adjusted after elution with neutralizing buffer (1 M Tris, pH 8.2). The flow-through (fraction 2) was transferred to sE-dimer-coated beads, incubated for 1 hour at RT on a rotator. Following incubation, the suspension was transferred to a chromatography column and centrifuged at 1,500 rpm for 10 seconds. The sE-dimer-coated beads were then washed with 500 µL of 1× PBS for 10 times, and elution buffer was added to collect the Abs fraction enriched for sE-dimer specific Abs (fraction 3). The flow-through (fraction 4) was transferred to sE-coated beads, incubated for 1 hour at RT on a rotator. After incubation, beads were transferred to the chromatography column and washed with 500 µL of 1× PBS for 10 times, and elution buffer was added to elute the anti-FL Abs (fraction 5). MIA confirmed the purification of each anti-E-protein epitope-specific Ab subset. The proportion of each Ab subset was calculated using the following formula: proportion of epitope-specific antibodies (%) = 100 × (MFI EDIII, sE, or total sE-dimer)/(MFI EDIII + sE + total sE-dimer). The isolation of each DENV-E mAbs was performed in two independent experiments, and data represent the mean ± s.e.m.

## RESULTS

### Optimization of MIA coupling for small antigens

**Fig 2:**
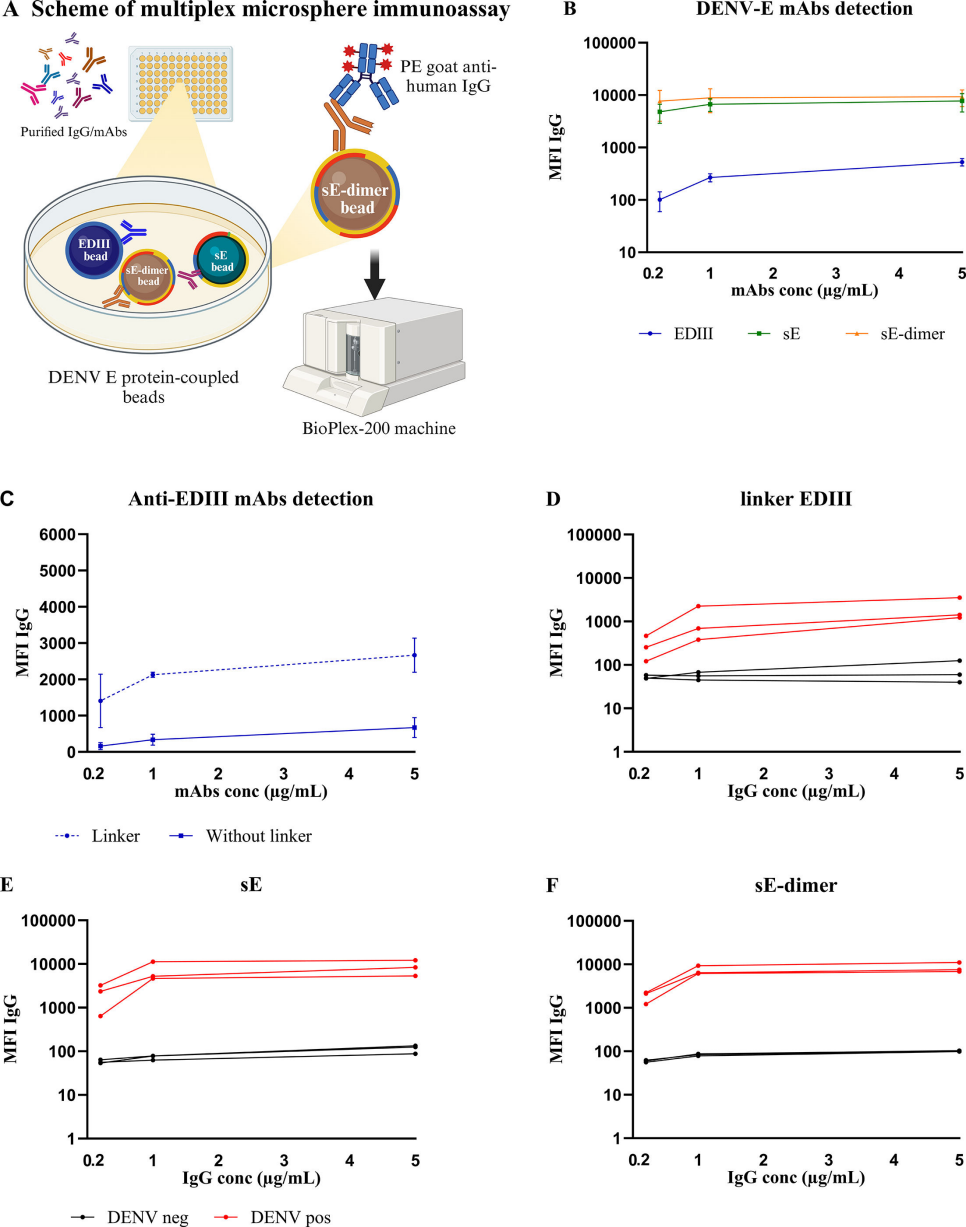
Validation of the coupling of DENV E recombinant proteins to the microsphere beads. (A) Scheme of MIA to detect anti-DENV E antibodies in human plasma. (B) Anti-DENV E mAb pool (containing mAb clone C8, 1A1D-2, 1A5) was serially diluted and tested for binding to EDIII (blue line), sE (green line), and sE-dimer (orange line) coupled beads. (C) Site-specific-biotinylated EDIII proteins were loaded onto streptavidin-coupled beads to generate a linker. EDIII-coupled beads with (dashed line) and without (solid line) linker were compared to detect anti-EDIII Abs in the anti-DENV E mAbs pool. Mean ± standard error of the mean (s.e.m). are shown of two independent experiments. To determine the threshold (D–F), three DENV IgG seropositive (red lines) and three DENV IgG seronegative (black lines) human plasma samples were used to determine the positivity threshold of anti-EDIII, anti-sE, and total anti-sE-dimer Abs. All data were expressed as MFI per 50 beads.

First, we coupled the recombinant proteins (EDIII, sE, sE-dimer) to the beads at concentrations from 2 to 10 µg per reaction using amine-based coupling. Protein binding was verified using a PE labeled anti-His-tag Ab. At 5 µg per reaction (EDIII = 263 pmol, sE = 100 pmol, and sE-dimer = 50 pmol), beads were saturated for all three antigens, and this concentration was chosen for the assay (Fig. S1). With these coupled beads and detection strategy (Fig. 2A) we were able to detect binding of pooled anti-DENV-E mAbs (clone 1A1D-2, C8, and 1A5, mimicking polyclonal IgG) at various concentrations ranging from 0.2 to 5 µg/mL to all three antigens (Fig. 2B). However, the signal for anti-EDIII Abs was low (<1,500 MFI at 5 and 10 µg of antigen) compared to the signals for anti-sE Abs (average 2,319 MFI at 5 and 10 µg of antigen) and total anti-sE-dimer Abs (average 2,280 MFI at 5 and 10 µg of antigen). Since this may be due to poor exposure of the epitopes in the small protein on the surface of the beads, we adapted the coupling strategy by introducing a linker between the beads and the EDIII antigen by first coupling the beads to streptavidin and subsequently by adding C-terminally biotinylated EDIII, taking advantage of the strong affinity between streptavidin and biotin, as reported previously (41). As a result, the MFI signal of anti-EDIII Abs detected using the linker increased 20-fold (Fig. 2C). In contrast, the signal decreased when using the linker strategy to couple sE and sE-dimer protein (Fig. S2). Following this, we tested all beads (linker-EDIII, sE, sE-dimer) for binding with total IgG isolated from DENV seropositive and DENV seronegative human plasma (Fig. 2D through F). The seropositive samples reached the binding equilibrium at an IgG concentration of 1 µg/mL. The average signal of DENV seronegative samples was 56 (SD = 11) MFI (EDIII), 73 (SD = 9) MFI (sE), and 83 (SD = 5) MFI (sE-dimer) at an IgG concentration of 1 µg/mL. Based on these results, we determined the threshold for positivity for human IgG at (mean + 3 standard deviation [SD]) which results in 100 MFI at an IgG concentration of 1 µg/mL.

### Assay performance validation

**Fig 3:**
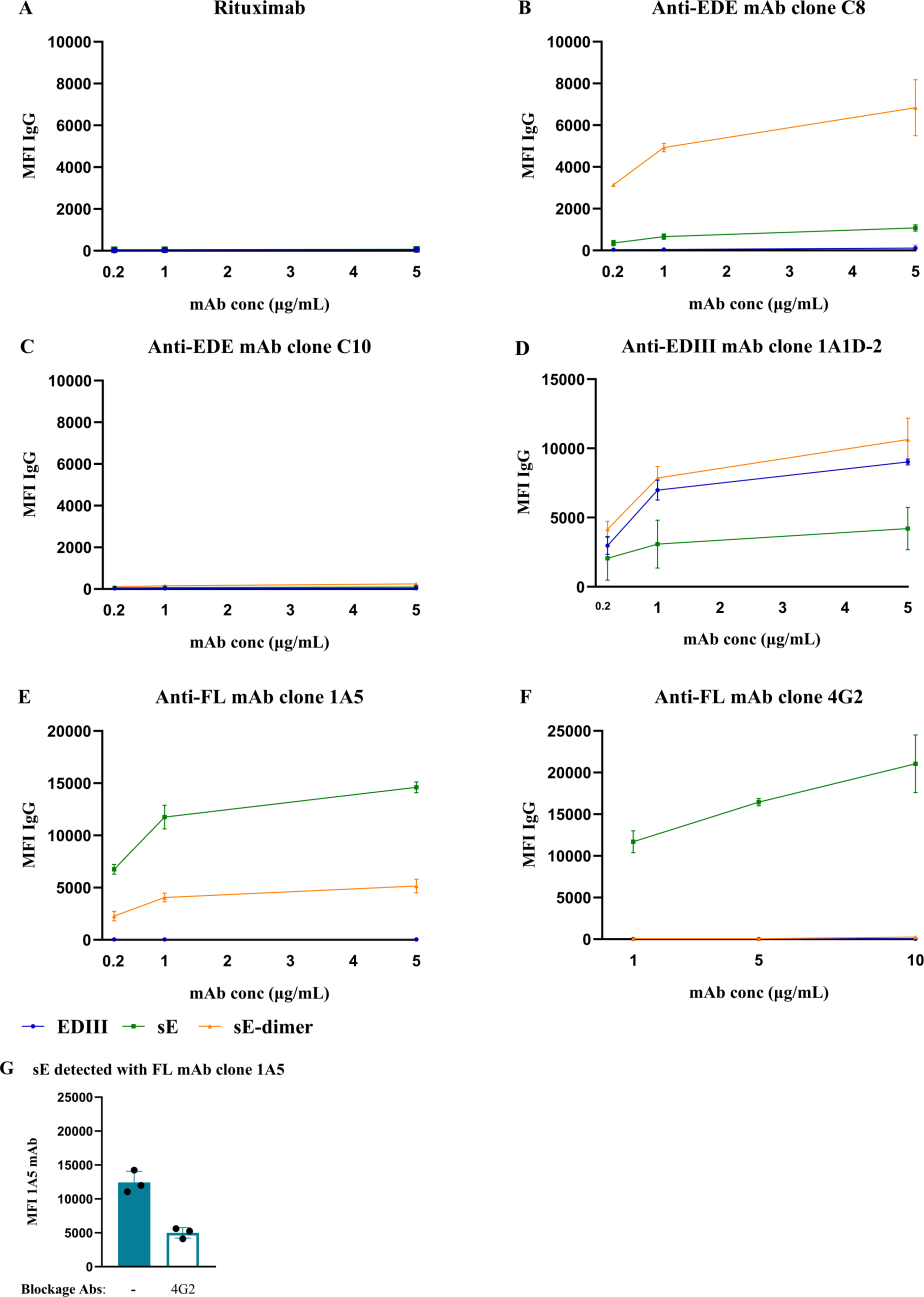
Detection of E-protein epitope-specific mAbs by MIA. (A) Rituximab, an anti-CD20 human mAb, was used as a negative control, and well-characterized anti-DENV E (B) mAb clone C8, (C) mAb clone C10, (D) mAb clone 1A1D-2, (E) FL-specific mAb clone 1A5, and (F) FL-mAb clone 4G2 were used to confirm binding specificity. (G) To determine anti-4G2-like Abs, mouse mAb clone 4G2 was used at a concentration of 10 µg/mL to block the FL epitope in sE. Mean ± standard error of the mean (s.e.m). are shown of two independent experiments.

To evaluate the assay performance, we measured the binding of various anti-E mAbs (human mAbs clone 1A1D-2, C8, C10, 1A5, mouse mAb clone 4G2) individually, as well as the anti-human CD20 mAb (Rituximab) as negative control. As expected, there was no binding of Rituximab to the antigen-coupled beads (Fig. 3A). In addition, mAb clone C8, which is an EDE-specific mAb binding to quaternary epitopes on the DENV virion, showed the strongest binding to sE-dimer coupled beads compared to sE and EDIII coupled beads (Fig. 3B) (40). MAb clone C10 also is an EDE-specific mAb, but C10 binding to the sE-dimer of DENV2 involves a distortion of the sE-dimer which is probably impaired in the cystine-locked sE-dimer (6, 30) . Indeed, the mAb clone C10 did not bind to sE-dimer coupled beads (Fig. 3C). The mAb clone 1A1D-2, previously reported to target EDIII, indeed bound to all three antigens (Fig. 3D) (37). The FL-specific mAb clone 1A5 bound preferentially to sE-coupled beads, confirming the exposure of the FL epitope in the recombinant sE-protein but not in the stabilized sE-dimer (Fig. 3E). Mouse mAb clone 4G2, a pan-flavivirus mAb targeting the FL epitope, bound exclusively to sE-coupled beads (Fig. 3F).

To further validate the detection strategy for E-protein epitope-specific Abs, we investigated the efficacy of FL epitope blocking by anti-FL mouse mAb clone 4G2. Indeed, pre-incubation with mouse mAb clone 4G2 can partially block the FL epitope leading to reduced binding of FL-specific 1A5 Ab (Fig. 3G).

### Determination of E-protein epitope-specific Abs in patient plasma

**Fig 4:**
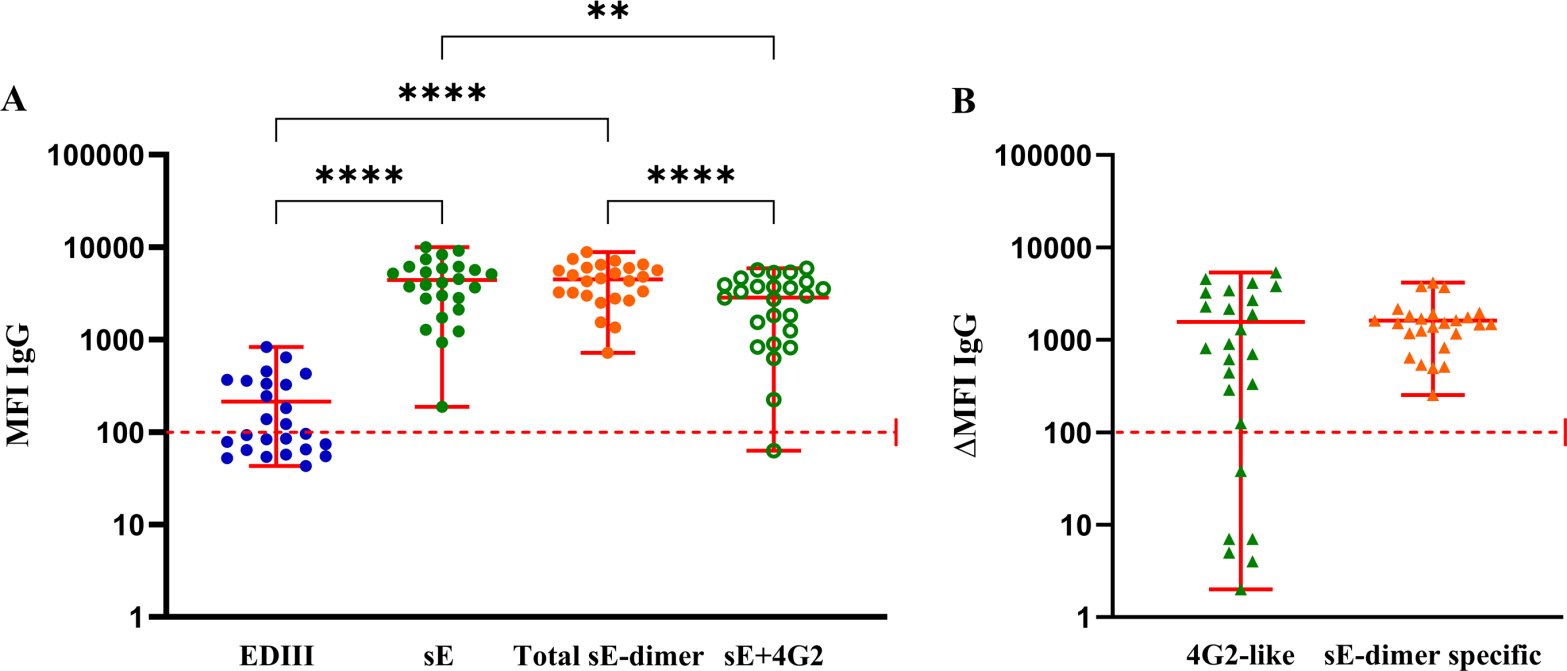
Detection of E-protein epitope-specific antibodies in human plasma by MIA. Detection of E-protein epitope-specific Abs using MIA in plasma of dengue fever patients (*n* = 25) obtained 4-6 days after onset of symptoms. All patients had confirmed secondary infection with DENV2. (A) The binding of Abs against EDIII (blue dots), sE (green dots), total sE-dimer (orange dots), and sE pre-blocked with mouse mAb clone 4G2 (green circles) were reported as MFI. (B) 4G2-like Abs (green triangles) are measured by subtracting the MFI signal of antibodies bound to sE pre-incubated with mouse mAb clone 4G2 from the total signal of antibodies bound to sE. sE-dimer specific Abs (orange triangles) were quantified by subtracting the MFI signal of antibodies bound to sE pre-incubated with mouse mAb clone 4G2 from the antibodies bound to sE-dimer protein. The data is shown as ∆MFI IgG. Each dot/circle/triangle represents an individual, and whiskers show maximum, minimum, and mean values. The dotted red line represents the threshold for positivity, The Wilcoxon test was used to compare two groups, while the Friedman test was used to compare multiple groups (*P < 0.05; **P < 0.01, ***P < 0.001; ****P < 0.0001).

We used this strategy to determine E-protein epitope-specific Abs in human samples (Table 1). Polyclonal total IgG from individuals with secondary DENV2 infection were tested. Anti-EDIII, anti-sE, and total anti-sE-dimer Abs were detectable. The MFI signal of anti-sE Ab subset for sE-coupled beads significantly decreased when pre-incubated with mouse mAb clone 4G2 (Fig. 4A). Relative abundance of anti-4G2-like Abs (representative of anti-FL Abs) are measured by subtracting the MFI signal of the Abs bound to sE incubated with mouse mAb clone 4G2 from the total signal of Abs bound to sE. Similarly, Abs targeting quaternary epitopes comprised of the sE-dimer surface (sE-dimer specific Abs) were quantified by subtracting the MFI signal of the Abs bound to sE incubated with mouse mAb clone 4G2 from the Abs bound to total sE-dimer (Fig. 4B). Taken together, we developed a novel serological multiplex immune assay to detect and quantify E-protein epitope-specific Abs in plasma from DENV-infected humans.

**TABLE 1:** Recombinant DENV E-proteins with their corresponding specific antibody binding and antibody name^*a*^

Antibody name	Protein	Antibody bound	Antibody not bound
Anti-sE Abs	sE	FL + EDI + EDII + EDIII	sE-dimer specific
sE – 4G2 like Abs	sE + 4G2	EDI + EDII + EDIII	sE-dimer specific + FL
Anti-EDIII Abs	EDIII	EDIII	EDI + EDII + FL + sE-dimer specific
Total anti-sE-dimer Abs	sE-dimer	sE-dimer specific + EDI + EDII + EDIII	FL
sE-dimer specific Abs	sE-dimer – (sE + 4G2)	Quaternary epitopes	FL, EDI, EDII, EDIII

^*a*^ sE protein consists of EDI, EDII, EDIII, and FL epitopes which are accessible for antibody binding, whereas the sE-dimer protein exposes EDI, EDII, EDIII, and quaternary epitopes.

### Purification of E-protein epitope-specific Abs from mAbs and human samples

**Fig 5:**
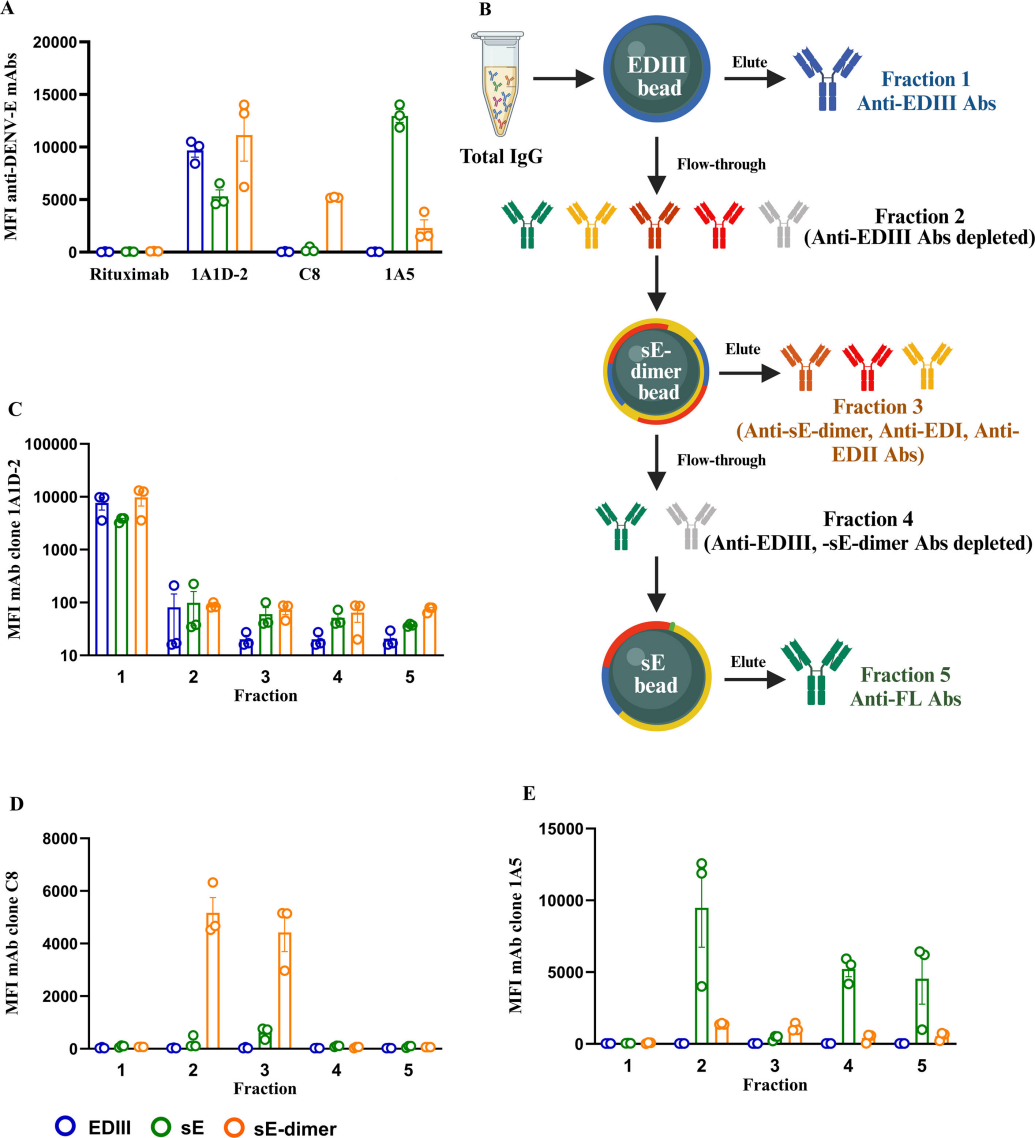
Confirmation of purification of E-protein epitope-specific Abs using mAbs. (A) The binding properties of mAbs (Rituximab, 1A1D-2, C8, and 1A5) before isolation were assessed against DENV E-proteins. (B) Isolation strategy. C-terminal biotinylated EDIII, sE, and sE-dimer proteins from DENV2 were coupled to streptavidin-resin beads for isolating E-protein epitope-specific Abs. Ab fractions were selected based on their specificity, employing both negative and positive selection techniques. (C–E) The purity of each fraction obtained from the isolation of anti-EDIII clone 1A1D-2 (C), anti-EDE specific mAb clone C8 (D), and anti-FL mAb clone 1A5 (E) was evaluated by MIA. Blue indicates the MFI of binding to the EDIII-coupled beads, green indicates the MFI of binding to the sE-coupled beads, and orange indicates MFI of binding to the sE-dimer-coupled beads. Mean ± standard error of the mean (s.e.m) are shown of two independent experiments.

We next designed a strategy to isolate E-protein epitope-specific Abs. The sequence of isolating epitope-specific Abs was designed to align with the hierarchical specificity and structural accessibility of the antigens (EDIII, sE-dimer, FL, sE) to minimize interference from overlapping epitopes and ensure a precise segregation of Ab subsets for subsequent functional analysis. To validate the purification strategy for isolating E-protein epitope-specific Abs, we purified well-characterized human anti-E mAbs (Fig. 5A and B). The binding properties of each mAb against DENV E-proteins before isolation were tested by MIA, and the amount of mAbs bound to each DENV E-protein were detectable according to their specificity (Fig. 5A). After isolation, the purity of each fraction was assessed by quantifying their binding to the different DENV E-proteins by MIA (Fig. 5C through E). Fraction 1 of mAb clone 1A1D-2, bound to all E-proteins. In fraction 2, the depletion of anti-EDIII Abs was complete as this fraction showed no binding to any proteins (Fig. 5C). From the isolation of EDE-specific mAb clone C8, fraction 3 bound preferentially to the sE-dimer coupled beads, while fraction 4 did not bind to sE-dimer beads (Fig. 5D). For anti-FL mAb clone 1A5, fraction 5 displayed binding exclusively to sE which is the only antigen containing the FL epitope (Fig. 5E). In summary, E-protein epitope-specific Abs can be successfully isolated using this strategy.

**Fig 6:**
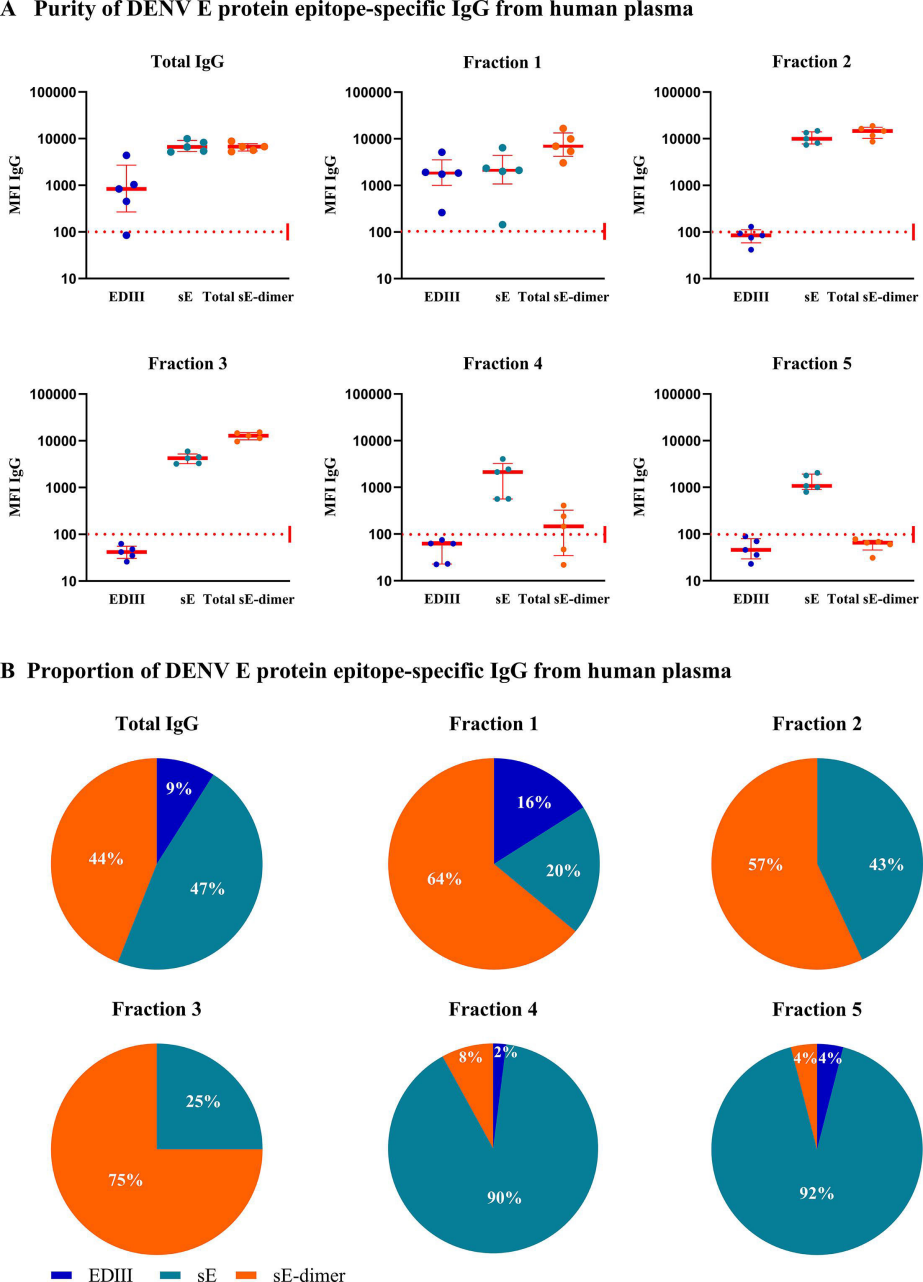
Purification of E-protein epitope-specific Abs from human plasma. Total IgG isolated from five DENV2 seropositive individuals was subjected to the Ab isolation strategy. (A) The IgG binding properties in each sample pre- and post-isolation were tested against all DENV E-proteins. Each dot represents an individual sample and is reported as MFI. Whiskers show max, min and mean value. The dotted red line represents the threshold for positivity. (B) Proportion of each E-protein epitope-specific Ab subset was calculated using the following formula: 100 × (MFI EDIII, sE, or total sE-dimer)/(MFI EDIII + sE + total sE-dimer).

We further assessed the isolation strategy using total IgG isolated from plasma obtained at day 3 after hospitalization of five individuals undergoing a secondary DENV2 infection (Fig. 6). Initial binding properties of IgG against DENV E-proteins were tested by MIA, revealing positive responses for all DENV E-proteins across all individuals, except one individual where anti-EDIII antibodies could not be detected. After purification on EDIII-coupled beads, fraction 1 (anti-EDIII antibodies) bound to all proteins, while fraction 2 (EDIII-depleted antibodies) bound to both sE and sE-dimer antigens but not EDIII, confirming fraction 1 as comprising anti-EDIII-specific Abs. In contrast, fraction 3 (EDIII depleted, total anti-sE-dimer Abs) preferentially bound to sE-dimer over sE and did not bind EDIII. Additionally, fraction 5 exclusively bound sE, indicating anti-FL-specific Abs (Fig. 6A). We also quantified the distribution of each E-protein epitope-specific Ab subset in each fraction (Fig. 6B). In conclusion, we have successfully purified anti-EDIII- and anti-FL-specific Abs and enriched a fraction of sE-dimer specific Abs from human plasma (Fig. S3).

## DISCUSSION

Over the last two decades, DENV vaccine development has faced significant challenges, primarily attributed to inadequate understanding of the immunological factors associated with protection (42, 43). The majority of investigations used total serum or plasma to describe the Ab response after natural DENV infection with assays incorporating whole DENV particles or recombinant E-proteins as antigens, such as the hemagglutination inhibition assay, plaque reduction neutralization assay, or ELISA (18, 27, 44, 45). Yet, this approach is unable to decipher and measure the subsets of the anti-DENV Ab response that target different E-protein epitopes, which might be better correlates of protection.

Therefore, we developed a multiplex MIA to measure Abs targeting EDIII, FL, and sE-dimer specific epitopes. Indeed, microsphere-based assays with a fluorescence readout are highly sensitive, display a large dynamic range, and a small amount of sample volume is needed compared to conventional ELISAs (46–49). We determined that 5 µg protein per coupling reaction was optimal to bind saturating amounts of antigen on the beads and that a biotin-streptavidin-based linker at the C-terminal side was able to enhance the anti-EDIII Abs signal. This indicates that coupling the small EDIII antigen (19 kDa) directly to the beads might lead to steric hindrance, which blocks access to EDIII Ab binding sites. Introducing a linker between the beads and EDIII creates space between the antigens themselves as well as between the antigen and the bead surface that increases accessibility of Ab to bind EDIII. Alternatively, or complementarily, epitope accessibility is enhanced due to the uniform orientation of the recombinant EDIII. A similar linker strategy has been successfully applied in a previous study (50). The MFI signal of the total IgG derived from seropositive plasmas were saturated at a total IgG concentration of 1 µg/mL, and the threshold for positivity was set at 100 MFI based on DENV seronegative samples.

In our study, we designed a strategy for the quantification and purification of DENV2 E-protein epitope-specific Abs in human plasma. MAb clone C8, which binds quaternary epitopes on the sE-dimer surface (40), showed the highest binding to sE-dimer coupled beads, confirming that our assay is able to detect sE-dimer specific Abs. However, mAb clone C10, another mAb targeting the quaternary epitopes on sE-dimer, did not show any binding to the stabilized dimer as previously shown by ELISA (30). This difference is probably due to the different binding of these two EDE-specific mAbs to the DENV2 sE-dimer, in particular C10 binding involves a distortion of the DENV2 sE-dimer which is probably impaired in the cystine-locked sE-dimer (PDB:7A37) (6). This distortion occurs in the case of binding of C10 to sE-dimers of DENV2, DENV3, and DENV4, but not for DENV1 or ZIKV sE-dimers. Thus, our DENV2 sE-dimer antigen will be able to capture most of the EDE-specific Abs but miss the ones similar to C10, which is a special case (30). While our assay measures Abs binding to the sE-dimer, we cannot distinguish between antibodies bound to the EDE epitope or other quaternary epitopes on the sE-dimer.

In addition, the mutated amino acid L107, located in the FL, is part of the epitope recognized by mAbs 4G2 and 1A5 (FL-specific Abs) (30, 36, 51). The absence of binding by these Abs for the sE-dimer antigen may be due to the presence of this mutation and not to the non-exposure of the FL in the sE-dimer antigen. Nevertheless, structural and functional studies (absence of insertion into membranes) have shown that the FL is hidden in the sE-dimer construct (30). The mAb clone 1A1D-2 bound to all three antigens, in line with the fact that this mAb binds to EDIII protein from DENV2 2 (37).

As expected, pre-incubation of sE with mouse mAb clone 4G2 masks the FL epitope as subsequent binding of the humanized FL-specific Ab clone 1A5 was abrogated. This blocking strategy allowed us to measure and calculate the amount of 4G2-like Abs, which are the prototype of FL Abs. Moreover, subtracting the binding of total sE-dimer Abs with sE pre-incubated with mouse mAb clone 4G2 allowed for the quantification of sE-dimer specific Abs in human plasma. Here, we use sE with 4G2 pre-incubation because it selectively depletes all similar Abs with the exception of the sE-dimer specific Abs, and therefore will allow for the quantification of sE-dimer specific Abs (see Table 1).

In order to fully characterize the functional properties of DENV E-protein epitope-specific Abs in human plasma, we have developed a strategy to isolate these antibodies. Using DENV E-derived proteins, anti-DENV E mAbs, and DENV seropositive plasma, we determined the optimal isolation strategy. This approach was effective for isolating anti-EDIII and anti-FL Abs and enriched the fraction of sE-dimer specific Abs from human plasma. This strategy will allow for the detailed functional characterization of these Ab subsets and the evaluation of their involvement in protection and/or pathogenesis of dengue disease in future studies.

The limitations of our study are related to the detection of some E-protein epitope-specific Abs. Firstly, we use E-derived proteins from DENV2 as proof of concept for future studies, as this is the main circulating serotype in Cambodia over the last 3 years and provides therefore a representative model for the study. However, future studies incorporating antigens from other serotypes will be valuable to validate and expand our findings. In addition, the FL-specific Abs we detected were limited to those targeting the same epitope as the mAb clone 4G2. Other FL Abs might target slightly different epitopes, but these will not be quantified with our strategy as we use 4G2 to block the FL epitope. However, mAb clone 4G2 remains the best characterized anti-FL Ab cross-reacting between all four DENV serotypes and even between different flavivirus sero-complexes (14, 15, 52). The quantification of 4G2-like Ab will give important insights into the anti-E Ab response. Moreover, the stabilization of the sE-dimer protein by introducing additional disulfide bridges affected the C10 binding site, limiting the detection of C10-like Abs (30).

Conclusively, a methodology has been developed for the identification and quantification of anti-EDIII, anti-sE, total anti-sE-dimer, anti-FL, and sE-dimer specific Abs in plasma from DENV-infected patients, which now can be employed to interrogate the abundance of E-protein epitope-specific Abs in different patient cohorts. Moreover, we can successfully isolate anti-EDIII and anti-FL Abs from human plasma, and we are able to enrich sE-dimer specific Abs paving the way for subsequent downstream applications. Further studies will employ this strategy to further characterize the functional properties of anti-EDIII, anti-FL, and sE-dimer specific Abs by using cell-based assays such as neutralization and ADE assays to increase understanding of the role of different E-protein epitope-specific antibodies in dengue disease pathogenesis.

## SUPPLEMENTAL MATERIAL

Supplemental figures msphere.00961-24-s0001.docx Figures S1 to S3.

ASM does not own the copyrights to Supplemental Material that may be linked to, or accessed through, an article. The authors have granted ASM a non-exclusive, world-wide license to publish the Supplemental Material files. Please contact the corresponding author directly for reuse.
