# Supplementary material for: Toward a deeper understanding of dengue: novel method for quantification and isolation of envelope protein epitope-specific antibodies
Source: mSphere. 2025 Apr 11;10(5):e00961-24. doi: 10.1128/msphere.00961-24 (PMC12108060; doi:10.1128/msphere.00961-24)
Supplement: Supplemental figures — Figures S1 to S3. [file msphere.00961-24-s0001.docx]

# Supplementary Material

**Supplemental figure 1:** **Determination of optimal DENV E-protein concentration needed for bead coupling.** Different color-coded magnetic beads were coupled to distinct DENV E recombinant proteins (EDIII without linker, sE, sE-dimer). Proteins were coupled to the beads at a concentration ranging from 2-10μg per reaction, and bound protein was detected by using anti-His tag PE antibodies.

**Supplemental figure 2: Difference between coupling of sE and sE-dimer with or without linker in bead coupling:** Different color-coded magnetic beads were coupled with streptavidin, and site-specific biotinylated sE (A) and sE-dimer (B) proteins were loaded onto the streptavidin coupled beads to generate a linker. Beads coupled with sE and sE-dimer, with (dashed line) and without (solid line) linker were compared to detect anti-sE and total anti-sE-dimer Abs in the anti-DENV E mAbs pool. The experiment was run in triplicate, and bars show mean with standard deviation

**Supplemental figure 3:** **Comparison of the proportion of sE-dimer specific Abs before and after isolation**. The total anti-sE-dimer Abs signal before and after isolation was calculated as a proportion of sE-dimer specific Abs and shown as a percentage. Each dot represents an individual. Paired t-test was used to compare two groups (*P < 0.05; **P < 0.01, ***P < 0.00; ****P < 0.0001)
